# Targeting of dopamine transporter to filopodia requires an outward-facing conformation of the transporter

**DOI:** 10.1038/s41598-017-05637-x

**Published:** 2017-07-14

**Authors:** Shiqi Ma, Mary H. Cheng, Daryl A. Guthrie, Amy H. Newman, Ivet Bahar, Alexander Sorkin

**Affiliations:** 1 Department of Cell Biology, School of Medicine, University of Pittsburgh, Pittsburgh, Pennsylvania USA; 2 Department of Computational and Systems Biology, School of Medicine, University of Pittsburgh, Pittsburgh, Pennsylvania USA; 3 Medicinal Chemistry Section, Molecular Targets and Medications Discovery Branch, National Institute of Drug Abuse-Intramural Research Program (NIDA-IRP), National Institutes of Health, Baltimore, Maryland USA

## Abstract

Dopamine transporter (DAT) has been shown to accumulate in filopodia in neurons and non-neuronal cells. To examine the mechanisms of DAT filopodial targeting, we used quantitative live-cell fluorescence microscopy, and compared the effects of the DAT inhibitor cocaine and its fluorescent analog JHC1-64 on the plasma membrane distribution of wild-type DAT and two non-functional DAT mutants, R60A and W63A, that do not accumulate in filopodia. W63A did not bind JHC1-64, whereas R60A did, although less efficiently compared to the wild-type DAT. Molecular dynamics simulations predicted that R60A preferentially assumes an outward-facing (OF) conformation through compensatory intracellular salt bridge formation, which in turn favors binding of cocaine. Imaging analysis showed that JHC1-64-bound R60A mutant predominantly localized in filopodia, whereas free R60A molecules were evenly distributed within the plasma membrane. Cocaine binding significantly increased the density of R60A, but not that of W63A, in filopodia. Further, zinc binding, known to stabilize the OF state, also increased R60A concentration in filopodia. Finally, amphetamine, that is thought to disrupt DAT OF conformation, reduced the concentration of wild-type DAT in filopodia. Altogether, these data indicate that OF conformation is required for the efficient targeting of DAT to, and accumulation in, filopodia.

## Introduction

Dopamine (DA) is an essential neurotransmitter in the mammalian central nervous system; it is involved in reward-motivated behavior, motor control, cognitive capacities development and attention regulation^1, 2^. Synaptically-released DA is primarily cleared from extraneuronal space by the plasma membrane dopamine transporter (DAT)^3^. The rate of DA clearance by DAT controls the duration and amplitude of post-synaptic DA signaling. DAT is the principle target for abused psychostimulants such as cocaine and amphetamine (AMPH)^4^.

DAT belongs to the high-affinity, sodium- and chloride- dependent SLC6 transporter gene family, which also includes serotonin, norepinephrine, glycine and γ-aminobutyric acid neurotransmitter transporters^5^. Like other members of the family, DAT consists of 12 transmembrane helical segments (TM), with TM1-5 and TM6-10 forming pseudo-symmetrically inverted repeats^6^. A centrally located high-affinity primary substrate-binding site (S1) lined by TM1, 3, 6 and 8 binds the substrate (DA) and ions, before their translocation and release to the cytoplasm. Helices TM1 and TM6 are broken into two segments each, TM1a, TM1b, TM6a and TM6b, near the DA/ions binding site. Another substrate-binding site (S2) is located closer to the extracellular (EC) vestibule of DAT and formed by residues from TM1, 3, and 10, and the EC loops (EL) 2 and 4^7^.

It has been proposed that DAT conformation dynamically shifts between outward-facing (OF) and inward-facing (IF) states during the transport cycle^8^. An intracellular (IC) interaction network involving TM1a, TM5, TM6b, TM8, and the N-terminal segment (a.a. 1-65) has been found to play a role in regulating the conformational transitions in DAT^9–11^. In particular, the closure of the IC vestibule in the OF state of DAT is stabilized by the salt bridge R60 - D436 (TM8) and the tri-aromatic interactions between W63, F332 and Y335 (TM6b)^9–11^. Disruption of this IC inteaction network was observed to faciliate the structural transition from OF to IF in DAT^11^ and in the structural homolog, leucine transporter (LeuT)^12^. Mutations of IC networking residues have been predicted to shift the conformational equilibrium toward the IF state^13^, in which the EC vestibule becomes less accessible (to the EC environment) than does the IC vestibule (to the cytoplasmic environment). Molecular dynamic (MD) simulations have demonstrated that binding of DA or AMPH drives a structural transition toward the IF state of DAT^7, 10, 11, 14^, while inhibitors such as cocaine stabilize DAT in the OF state^15, 16^ through competitive binding to S1 site^14, 17, 18^. Similarly, the serotonin transporter (SERT) exhibits the same alternation between outward- and inward-facing states, driven by substrates and inhibitors^19^. Obviously, maintaining OF conformation is critical for the substrate uptake function of DAT, and the transition to the IF state is essential for substrate release. However, whether such conformation states (or their transitions) affect the subcellular distribution of DAT has not been elucidated.

DAT is exclusively expressed in dopaminergic neurons that have a highly complex morphology, with the somatodendritic compartment located in the midbrain and highly-branched and arborized axons projecting mainly to dorsal striatum and nuclear accumbens^2^. The highest density of DAT is detected in the presynaptic surface of axons in the striatum and the nuclear accumbens^20, 21^. The mechanisms responsible for targeting of DAT to axonal membranes are not understood. We have previously demonstrated that DAT is accumulated in filopodia in dopaminergic neurons and non-neuronal cells^22–24^. We proposed that the ability to accumulate in the highly-curved membranes of filopodia may be enabled by the same mechanism that is also responsible for DAT accumulation in dopaminergic axons whose dimensions and membrane curvature are similar to those of filopodia, especially during axonal branching^22–24^.

Filopodia are thin finger-like protrusions of the plasma membrane containing a uniform bundle of actin filaments^25^. They are involved in neurite outgrowth, axon branching and dendritic spine formation^26^. Filopodia formation begins with the negative (away from the cytoplasm) bending/protrusion of the membrane, that can be mediated by I-BAR (inverted-Bin-amphiphysin-Rvs) domain proteins such as IRSp53^27–29^. I-BAR domain supported membrane protrusions are further stabilized by actin filaments, which is necessary for efficient protrusion and a proper length of filopodia^27–29^. We have previously proposed that overexpression of DAT may also lead to filopodia formation. Based on the observation that DAT mutants with disrupted intramolecular interactions and diminished substrate transport activity were not enriched in filopodia, we hypothesized that proper functional conformation of the DAT molecule is necessary for its targeting to filopodia and/or filopodia-promoting activity^24^. In the present study, we directly examine the relationship between DAT filopodia targeting and DAT conformational states. We use MD simulations to determine the conformational state of two DAT mutants, and define the effects of cocaine and its fluorescent analog JHC1-64 on the conformation of these mutants. We then use several independent experimental approaches to control the conformational state of DAT and its mutant to demonstrate that the OF state of DAT is required for its enrichment in filopodia.

## Results

### Fluorescent cocaine analog JHC1-64 preferentially binds to filopodia-located DAT and its R60A mutant

To quantitatively compare the distribution of the wild-type (wt) DAT and non-functional DAT mutants on the plasma membrane, wt YFP-HA-DAT and its mutants R60A and W63A^23^ were transiently expressed in HEK293 and PAE cells. Transient expression of wt YFP-HA-DAT and its mutants was used to avoid clonal variations of stably expressing cells, and differences in expression levels of wt and mutant DATs in these clones. Various types of experiments yielded essentially similar results in HEK293 and PAE cells.

**Figure 1:**
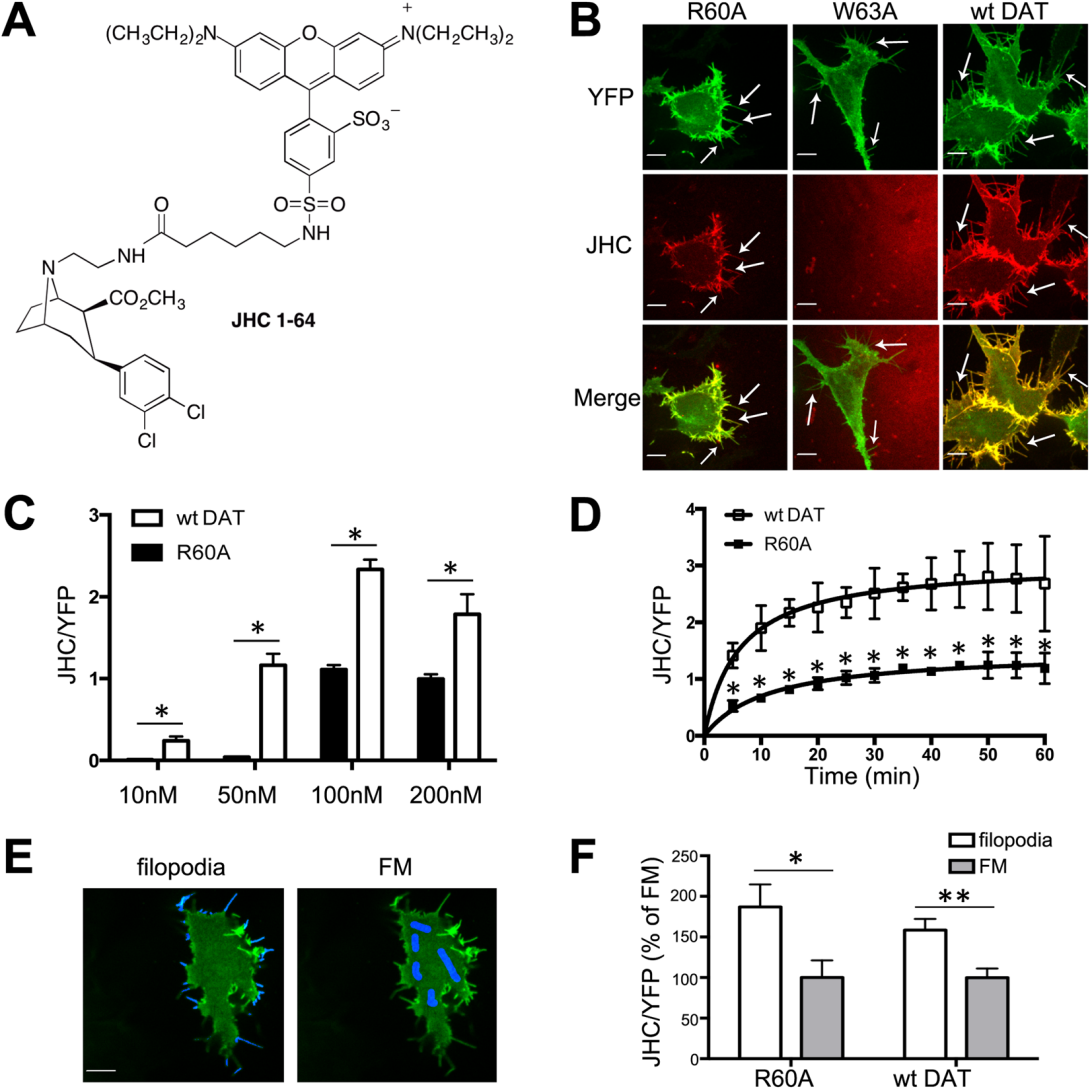
Preferential binding of JHC1-64 to wt DAT and its R60A mutant in filopodia and cell edges of HEK293 cells. (**A**) Chemical sstructure of JHC1-064^30^. (**B**) Cells transiently expressing wt YFP-HA-DAT, and R60A or W63A mutants of YFP-HA-DAT were incubated with 100 nM JHC1-64 at RT for 30 min. Live-cell imaging was performed through 515 nm (YFP, *green*) and 561 nm (JHC1-64, *red*) filter channels. Maximal projections of 5 z-planes are shown. Arrows point to examples of peripheral filopodia. Scale bars, 10μm. (**C**) YFP-HA-DAT or the R60A mutant were treated with 10-200 nM JHC1-64 for 30 min at RT. The ratio of JHC1-64 and YFP fluorescence intensities (designated JHC/YFP) was quantitated for whole cells as described in ‘Methods’. Bars represent mean values (±SEM; n = 3). (**D**) Cells were treated with 100 nM JHC1-64 as in (**B**), and the time dependence of the JHC/YFP ratio was determined by time-lapse imaging of YFP and JHC1-64. Mean values are presented (±SEM, n = 3). (**E**) Examples of the segmentation masks generated as described in “Methods” for quantification of the mean fluorescence intensities of peripheral filopodia (*left*) and non-filopodial membrane (flat membrane, FM; *right*) regions from YFP images obtained in experiments represented in (**B**). (**F**) Cells expressing YFP-HA-DAT or R60A were imaged after incubation with 100 nM JHC1-64 for 30 min at RT. The JHC/YFP ratio in filopodia and FM masks was quantitated in individual cells. Results are shown as mean values of JHC/YFP ratios relative to the ratios in FM mask in each individual cell (±SEM, n = 10). *p < 0.05, **p < 0.01.

JHC1-64 (Fig. 1A), a membrane-impermeable fluorescent cocaine analog, was used to detect surface YFP-HA-DAT^30^. It was previously shown that JHC1-64 does not alter the membrane mobility and trafficking properties of DAT^31^. Cells expressing YFP-HA-DAT or its mutants were incubated with 100 nM JHC1-64 for 30 min at room temperature (RT). Live-cell imaging was performed at RT to minimize the motility of highly dynamic filopodia during 3D image acquisition. As shown in Fig. 1B, JHC1-64 bound to the R60A mutant, albeit to a lesser extent than to wt YFP-HA-DAT. By contrast, no detectable binding of JHC1-64 to the W63A mutant was observed (Fig. 1B). JHC1-64 concentration of 100 nM was found to yield the best signal-to-noise fluorescence ratio in cells expressing wt YFP-HA-DAT or the R60A mutant (Fig. 1C). Quantification of the JHC1-64/YFP ratio during the time-course of JHC1-64 (100 nM) binding to wt YFP-HA-DAT and to the R60A mutant using single-cell imaging showed that binding to either DAT approached maximum after 20-30 min at RT, and that binding to the R60A mutant was 2-times less efficient than that to wt YFP-HA-DAT (Fig. 1D). Binding of JHC1-64 at 37 °C was much faster compared to that at RT (maximum binding to wt YFP-HA-DAT was reached at 1 min), while the pattern of JHC1-64 localization was identical to that observed at RT. No significant dissociation of JHC1-64 was observed after washing out JHC1-64 for at least 1-hr incubation at 37 °C, suggesting that JHC1-64 dissociation rate constant is low (data not shown).

Visual inspection of JHC1-64 localization revealed the striking preference of JHC1-64 to decorate filopodia and cell edges, areas of increased membrane curvature (Fig. 1B). Accumulation of both JHC1-64 and YFP-HA-DAT (indicative of the formation of JHC1-64:YFP-HA-DAT complexes) was most pronounced in filopodia emanating near cell-cell contacts. The ratio of the fluorescence intensities of JHC1-64 and YFP-HA-DAT, designated as JHC/YFP, was adopted as a measure of the relative concentration of JHC1-64:YFP-HA-DAT complexes (see “Methods”). To compare the JHC/YFP ratio in the filopodia to that in regions of the planar plasma membrane with the diffuse distribution of YFP-HA-DAT (FM, flat membrane), peripheral filopodia and FM masks were generated in each cell (illustrated in Fig. 1E). JHC/YFP ratio values were quantified in the peripheral filopodia because DAT density in dorsal filopodia is technically difficult to measure due to limited z-axis resolution. This analysis revealed that the concentration of JHC1-64:YFP-HA-DAT complexes was at least 2-fold higher in filopodia than in flat membrane regions. The same behavior was observed in cells expressing the R60A mutant (Fig. 1F). These data suggest that both wt YFP-HA-DAT and its R60A mutant either bind JHC-64 more efficiently in filopodia or move to filopodia after JHC1-64 binding.

### JHC1-64 and cocaine increase R60A concentration in filopodia

The observation of a high concentration of the JHC1-64-bound R60A mutant in filopodia prompted us to test whether JHC1-64 binding promotes targeting of this mutant to filopodia or whether those mutant transporters already located in the filopodia exhibit a higher propensity to bind JHC-64.

**Figure 2:**
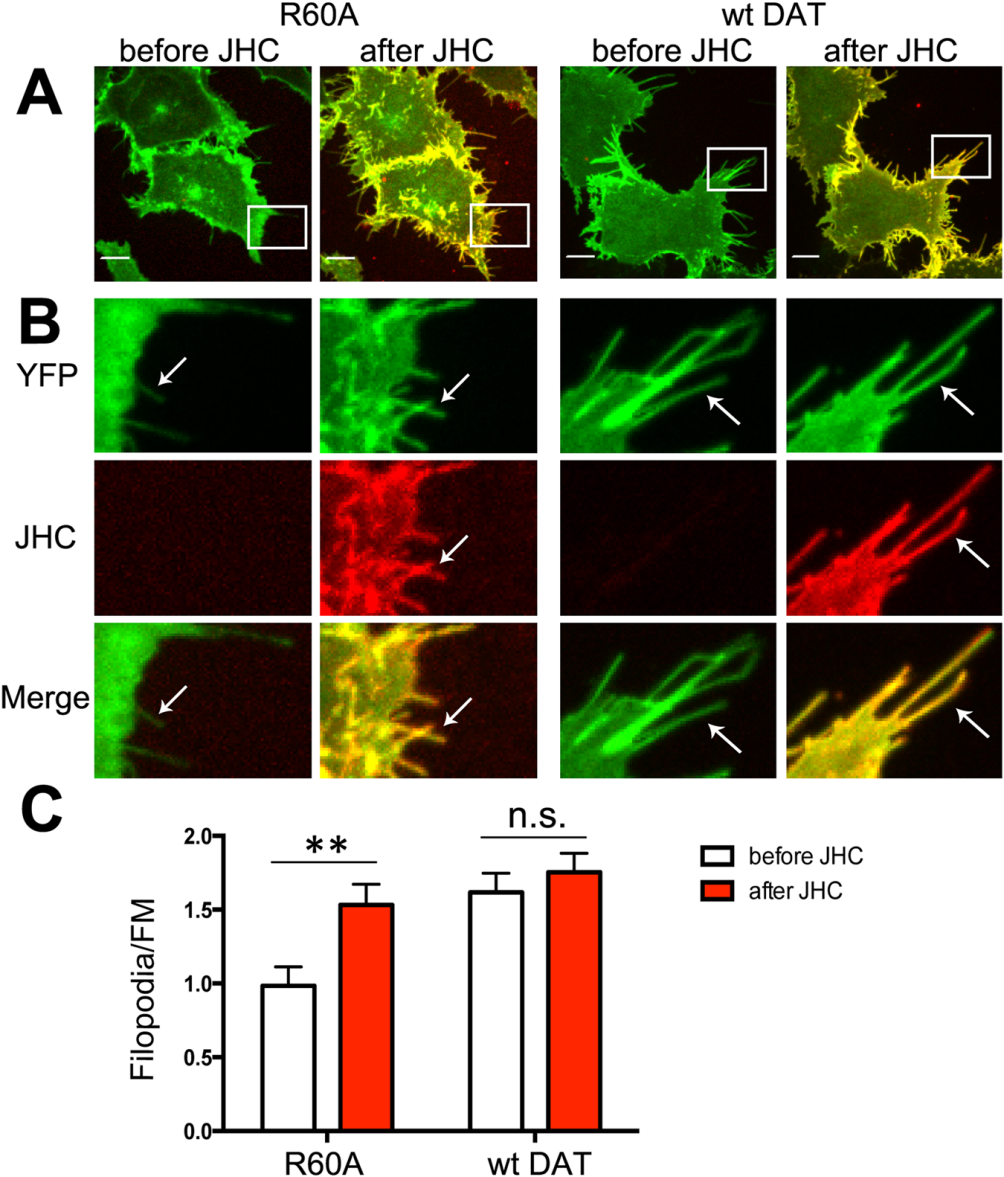
Binding of JHC1-64 increases the concentration of R60A in filopodia of HEK293 cells. (**A**) Cells transiently expressing YFP-HA-DAT or the R60A mutant were incubated with 100 nM JHC1-64 for 30 min at RT. Live-cell imaging was performed through 515 nm (YFP, *green*) and 561 nm (JHC1-64, *red*) filter channels. Maximal z-projections of 5 consecutive x-y-confocal planes are shown. Scale bars, 10μm. (**B**) Insets represent high magnification images of the regions marked by the white rectangle in (**A**). Arrows point to representative filopodia. (**C**) The Filopodia/FM ratios of mean intensities of the YFP fluorescence were calculated as described in the “Methods”. Results are shown as mean values (±SEM, n = 10). **p < 0.01.

To this end, we analyzed by 3D imaging the density of YFP-HA-DAT (mean fluorescence intensity per image voxel) in peripheral filopodia and non-filopodial regions of the plasma membrane in the same single HEK293 cells before and after incubation with JHC1-64 for 30 min at RT as described in “Methods”. These experiments demonstrated that binding of JHC1-64 increased the filopodia/FM ratio of the YFP fluorescence intensity in cells expressing the R60A mutant by 50%, suggesting an elevated targeting of R60A mutant to filopodia in the presence of JHC1-64 (Fig. 2). The distribution of wt YFP-HA-DAT in filopodia was not significantly affected by JHC1-64 (Fig. 2). Similar filopodia targeting effect of JHC1-64 on R60A (increase in the filopodia/FM ratio by ~30%) was observed in PAE cells (Supplementary Information; Fig. S1).

**Figure 3:**
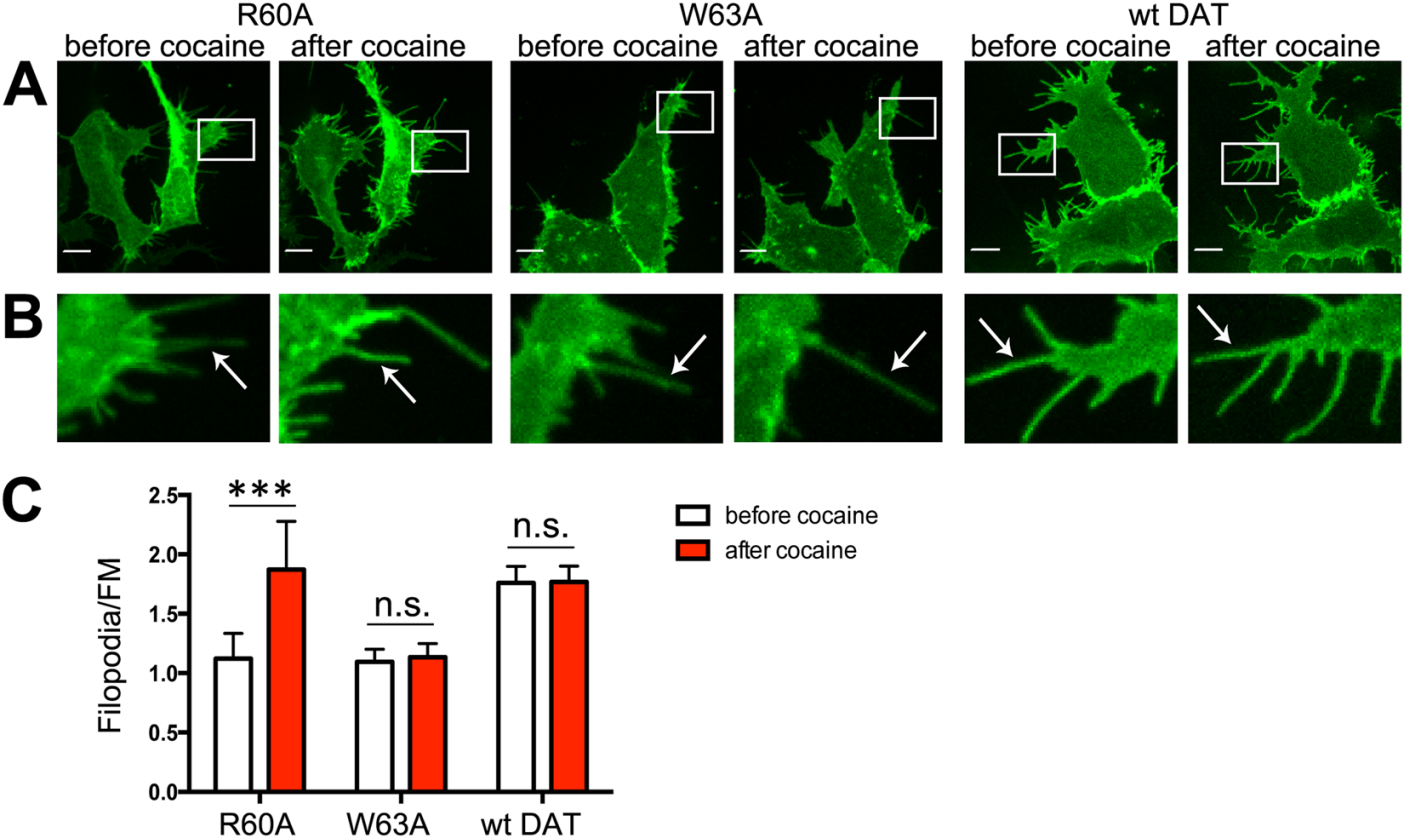
Binding of cocaine increases the concentration of R60A (but not W63A) in the filopodia of HEK293 cells. (**A**) Cells transiently expressing wt YFP-HA-DAT, or its R60A or W63A mutants were incubated with 10 μM cocaine for 30 min at RT. Live-cell imaging was performed through the 515 nm (YFP, *green*) filter channel. Maximal z-projections of 5 consecutive x-y-confocal planes are shown. (**B**) Insets represent high magnification images of the regions marked by the white rectangle in (**A**). Arrows point to representative filopodia. (**C**) The filopodia/FM ratios of mean intensities of the YFP fluorescence were calculated as described in “Methods”. Results are shown as mean values (±SEM, n = 10). ***p < 0.001.

We further tested whether unlabeled cocaine, that has slightly lower binding affinity (than JHC1-64) to DAT but is available for use in high concentrations allowing a maximum occupancy of surface DATs, would also cause the re-distribution of the R60A mutant toward filopodia. Consistently with the effects of JHC1-64, the filopodia/FM ratio in HEK293 cells expressing the R60A mutant increased by 60% after cell incubation with cocaine, whereas the subcellular distribution of wt YFP-HA-DAT did not change (Fig. 3A–C). Similar results were observed in PAE cells where the filopodia/FM ratio of the R60A mutant increased by 70% after cocaine treatment (Fig. S2). Cocaine treatment did not result in increased targeting to filopodia of the W63A mutant that does not bind JHC1-64, confirming that elevated filopodial targeting of R60A was mediated by cocaine through its binding to this mutant transporter (Fig. 3C).

**Figure 4:**
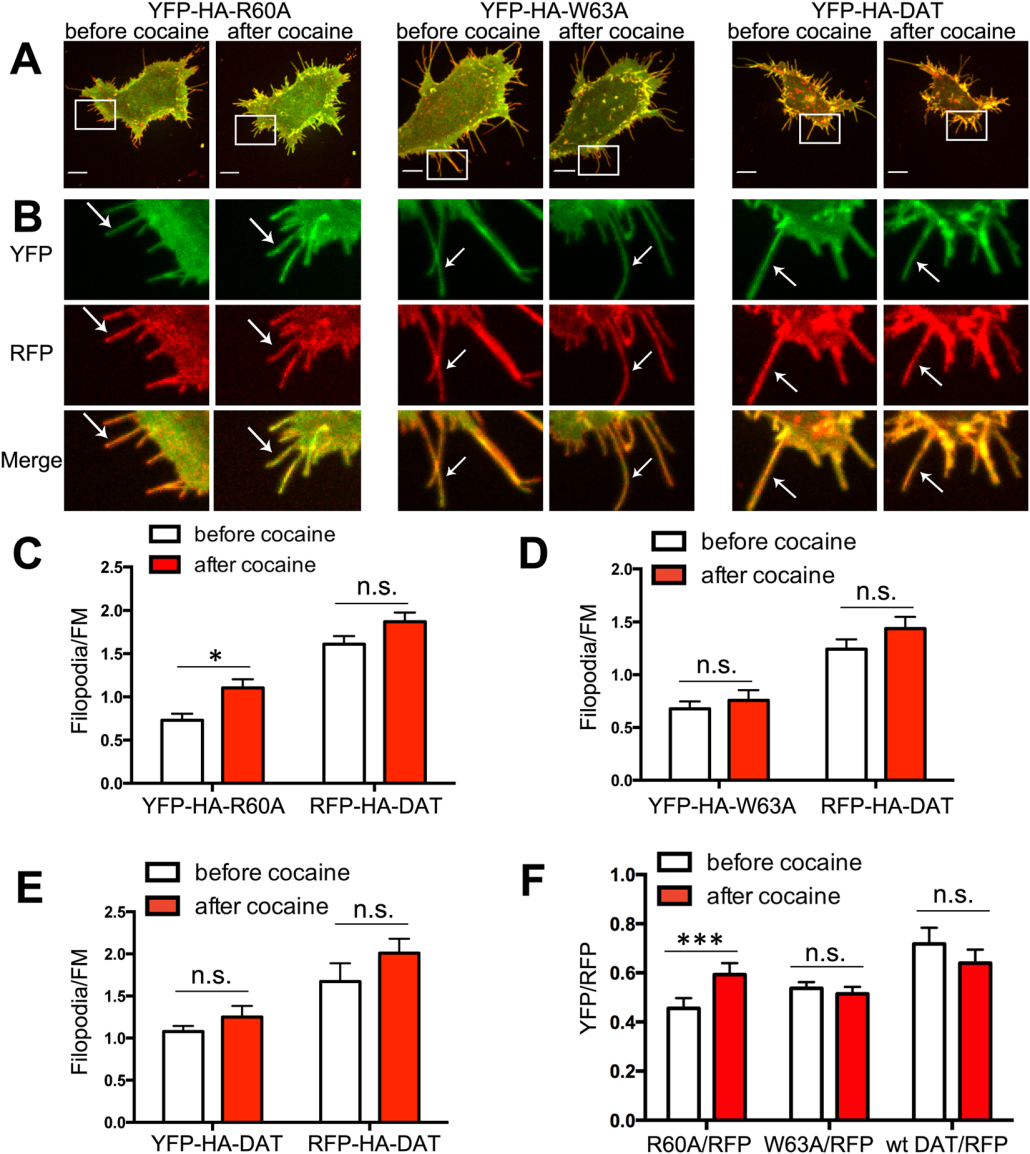
Binding of cocaine results in the enrichment of the R60A mutant relative to co-expressed wt DAT in the filopodia of HEK293 cells. (**A**) HEK293 cells co-expressing RFP-HA-DAT with YFP-HA-DAT, YFP-HA-DAT-R60A or YFP-HA-DAT-W63A were imaged through 515 nm (YFP, *green*) and 561 nm (RFP, *red*) filter channels before and after cocaine treatment (30 min at RT). Maximal z-projections of 5 consecutive x-y-confocal planes are shown. Scale bars, 10μm. (**B**) Insets represent high magnification images of the regions marked by the white rectangle in (**A**). Arrows point to examples of peripheral filopodia. (**C–E**) The Filopodia/FM ratios of YFP and RFP fluorescence intensities in cells co-expressing RFP-HA-DAT with YFP-HA-R60A (**C**), YFP-HA-W63A (**D**) or wt YFP-HA-DAT (**E**) were calculated in experiments exemplified in (**A,B**) as described in “Methods”. Results are shown as mean ± SEM, n = 10. (**F**) The YFP/RFP ratio in filopodia was calculated in experiments represented in (**A,B**). Results are shown as mean ± SEM, n = 10. *p < 0.05, ***p < 0.001. *p < 0.05, ***p < 0.001.

We further examined the effect of cocaine on the filopodia targeting of DAT mutants using an independent quantitative imaging approach: YFP-HA-DAT or its mutants were co-expressed with wt RFP-HA-DAT in HEK293 cells. This approach enabled direct comparison of the local densities and localization of wt and mutant DATs by measuring the ratio of YFP-HA-R60A and RFP-HA-DAT fluorescence intensities in filopodia and FM regions in single cells imaged before and after incubation with cocaine for 30 min at RT. YFP-tagged DATs were co-localized with RFP-HA-DAT (Fig. 4A,B). The Filopodia/FM ratio of the R60A mutant was increased upon binding of cocaine by ~2-fold (Fig. 4C), whereas the Filopodia/FM ratios of wt YFP-HA-DAT and the W63A mutant were not significantly changed (Fig. 4D,E). Calculation of the YFP/RFP ratios (see “Methods”) demonstrates that cocaine caused a relatively stronger enrichment of the R60A mutant than wt RFP-HA- DAT in filopodia (Fig. 4F). By contrast, the YFP/RFP ratio in cells expressing YFP-HA-W63A and RFP-HA-DAT was not increased by cocaine (Fig. 4F), consistent with the inability of W63A mutant to bind cocaine. The data in Fig. 4 are in agreement with the results presented in Figs 2 and 3, and altogether these measurements demonstrate that binding of either cocaine or its analog JHC1-64 favors localization of the R60A mutant to filopodia.

### Modeling of the binding of cocaine and JHC1-64 to wt- and mutant-DAT

**Figure 5:**
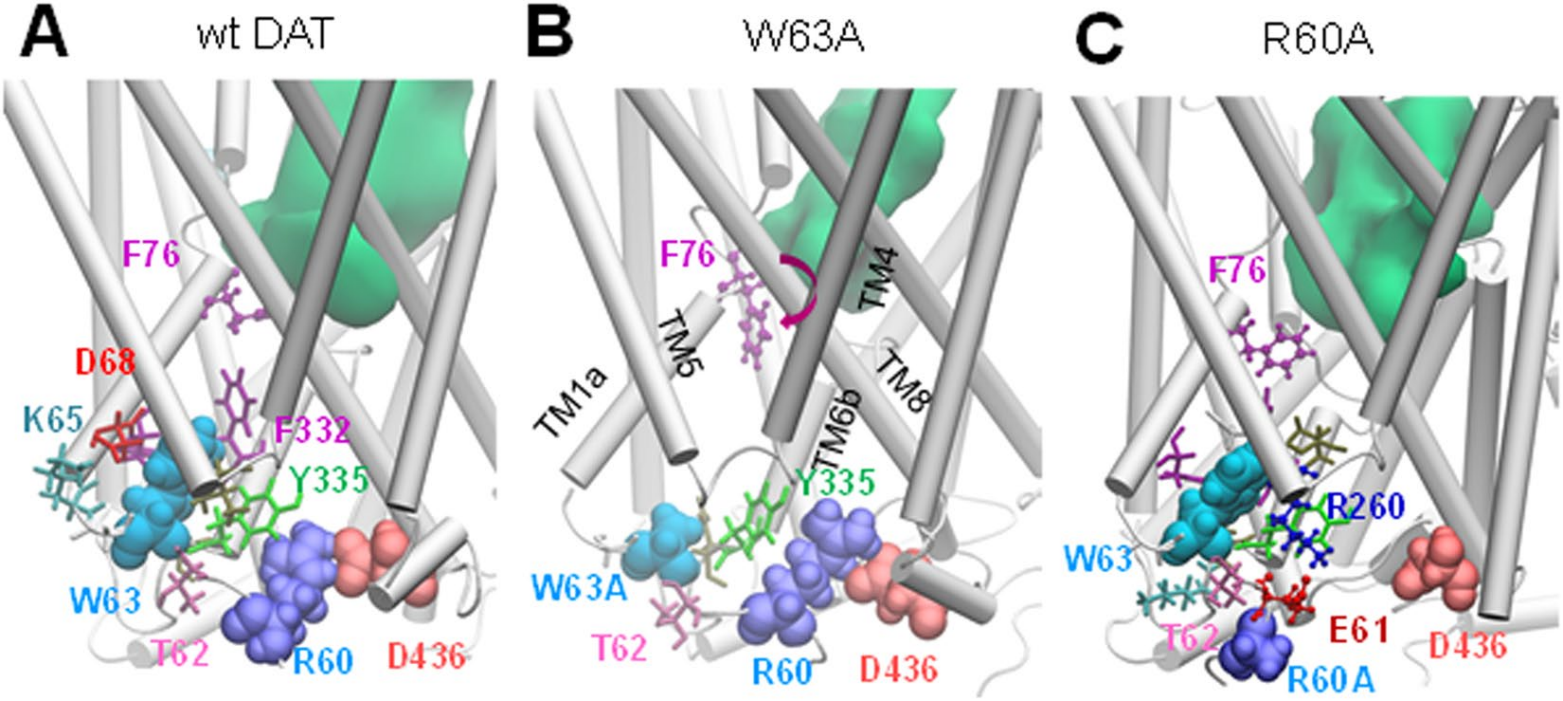
The IC interaction network that regulates the opening/closure of the IC vestibule and the transition between OF and IF states, shows distinctive dynamics in wt DAT and mutants W63A and R60A. (**A**) In wt DAT, the salt-bridge R60-D436 and close interactions between W63 and the F332 and Y335 stabilize substrate/sodium-binding site in the OF state. (**B**) In the mutant W63A, substitution of W63 by alanine breaks the stabilizing network of molecular interaction; F76 side chain dihedral angle *χ*_*1*_ rotates from ± 180° to −60°, leading to the opening of the IC vestibule. (**C**) Substitution of R60 by alanine breaks the salt-bridge R60-D436. Yet, alternative IC salt bridges form, such as R260-E61, which stably maintain the closed state of the IC vestibule. Residues at positions 60 and 63 are displayed in VDW format, as well as D436. In all diagrams, residues within 3 Å of W63 or W63A are shown in licorice format. Hydrated EC regions are indicated by *green* shaded areas.

To gain a mechanistic understanding of the effect of cocaine or JHC1-64 on the distribution of wt and mutant DATs on the plasma membrane (as demonstrated in Figs 1, 2, 3 and 4), a series of MD and docking simulations were performed. Within 100 ns MD simulations, we observed that the wt DAT and the mutants R60A and W63A exhibit distinctive conformational dynamics near the IC network of interactions, with different effects on the opening/closure of the EC and IC vestibules (Fig. 5). The substitution of alanine in W63A weakened the interactions between the TM1a, TM5 and TM6b segments, and altered the geometry at the substrate-/sodium-binding site: the side chain of F76 rotated away from the binding site (Fig. 5B), facilitating the opening of the IC vestibule, and enhancing the stability of the IF state, at the expense of the OF state. In contrast, the weakening of the IC interactions in the R60A mutant was compensated via re-distribution of salt bridges, e.g., formation of a new salt bridge, E61-K260 (Fig. 5C). This preserved the closed state of the IC vestibule, in favor of the opening of the EC vestibule. These simulations suggest that wt DAT and the R60 mutant maintain their access to the OF state, while W63A does not.

**Figure 6:**
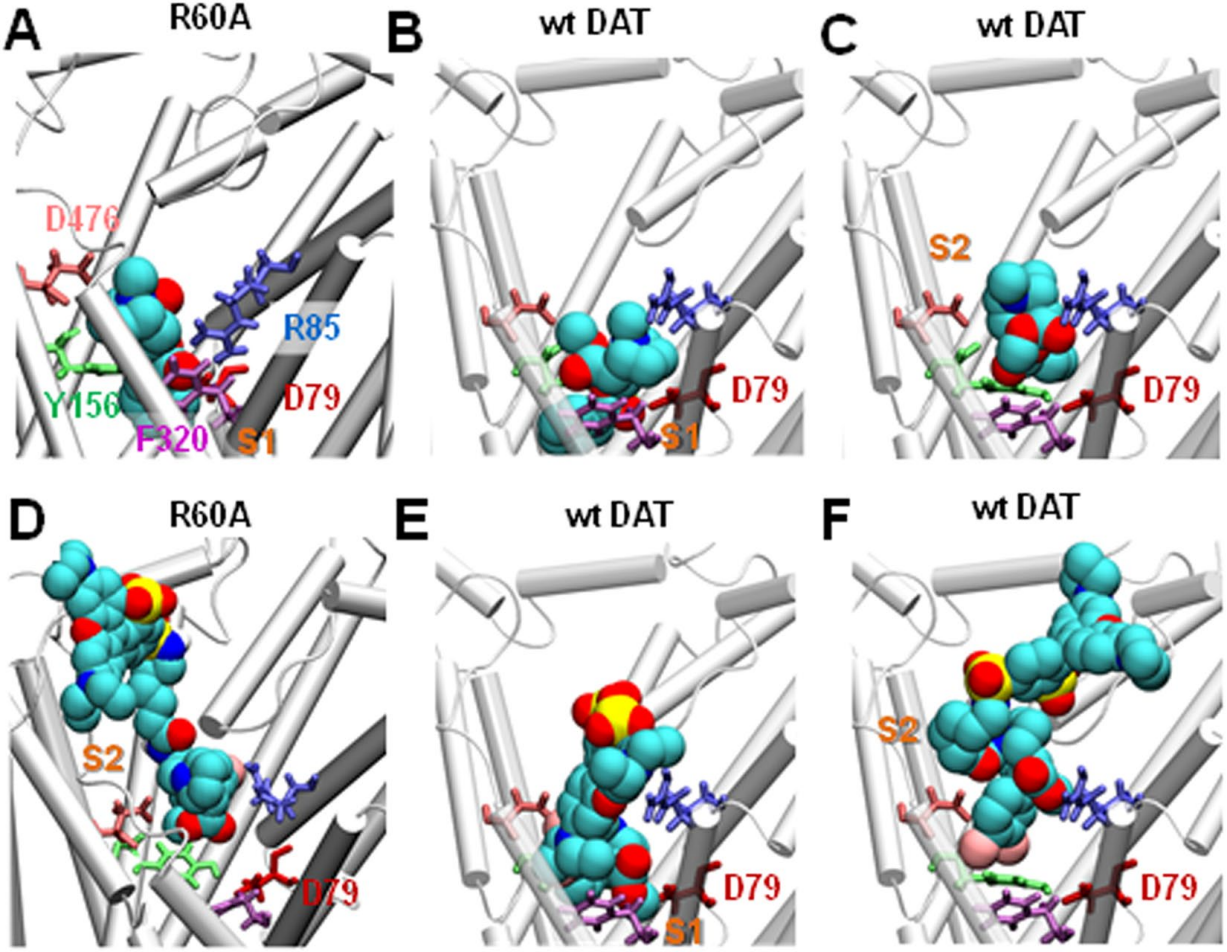
Cocaine and its analog bind and stabilize the outward-facing (OF) state to the wt DAT and the R60A mutant. (**A**) Cocaine binds near the S1 site of R60A in the OF state of the mutant, and closely interacts with D79, R85, Y156, F320 and D476. (**B,C**) Cocaine binds near the S1 (**B**) and S2 (**C**) sites of wt DAT in the OF state. (**D**) JHC1-64 binds near the S2 site of R60A and inserts toward the S1 site. **(E,F)** JHC1-64 binds to the vicinity of the S1 (***E***) and S2 (***F***) sites of wt DAT. *Cyan, blue, dark red, light red*, and *yellow* spheres represent the respective carbon, nitrogen, oxygen, chloride, and sulfur atoms of JHC1-64.

In our previous docking simulations^14^, cocaine was observed to bind the OF state of DAT at both the substrate-binding site S1 and the site S2 near the EC entrance which was proposed to serve as a first recognition site before inserting into the S1 site. Strikingly, the predicted binding pose of cocaine to site S1 showed close similarity to that resolved for the *Drosophila* DAT X-ray structure^17^. Furthermore, our prediction of cocaine binding to the S2 site in DAT^14^ coincides with an allosteric site resolved in the antidepressant-bound serotonin transporter^32^. Here, using the same docking protocol, we estimated the cocaine binding site in the R60A and W63A mutants. The most favorable model obtained for R60A was confined to the close vicinity of site S1, with a lower affinity (−5.0 ± 0.3 kcal/mol) (Fig. 6A) as compared to wt (−6.2 ± 0.3 kcal/mol) (Fig. 6B). In the W63A mutant, we did not observe cocaine binding inside the EC vestibule (data not shown), likely due to significant alteration of the binding site S1 and decrease of the EC vestibule (Fig. 5B). These data suggest that binding of cocaine can stabilize the R60A (but not the W63A mutant) in the OF state, similar to its action on the wt DAT^15, 16^.

To determine the binding properties of JHC1-64 in DAT, we simulated its docking to wt and mutant DATs (see “Methods”). The predicted binding poses of JHC1-64 to DAT (Fig. 6E,F) were comparable to those of cocaine in the OF state of DAT. Notably, JHC1-64 bound to both sites S1 (Fig. 6E; interacting with D79 at the lower portion of the EC vestibule) or S2 (Fig. 6F; at the upper portion of the EC vestibule). Like cocaine, JHC1-64 did not show any docking occupancy inside the EC vestibule of W63A (data not shown). On the other hand, JHC1-64 was able to bind deep inside the EC vestibule of the R60A (Fig. 6D), albeit with a lower binding affinity (−6.5 ± 1.05 kcal/mol) as compared to wt DAT (−8.0 ± 0.5 kcal/mol). These results were consistent with observations made in live-cell imaging experiments (Fig. 1). Overall, our MD simulations and docking analysis predict that binding of cocaine or JHC1-64 stabilizes the OF conformation of both wt DAT and the R60A mutant.

### Zinc increases the concentration of R60A in filopodia

**Figure 7:**
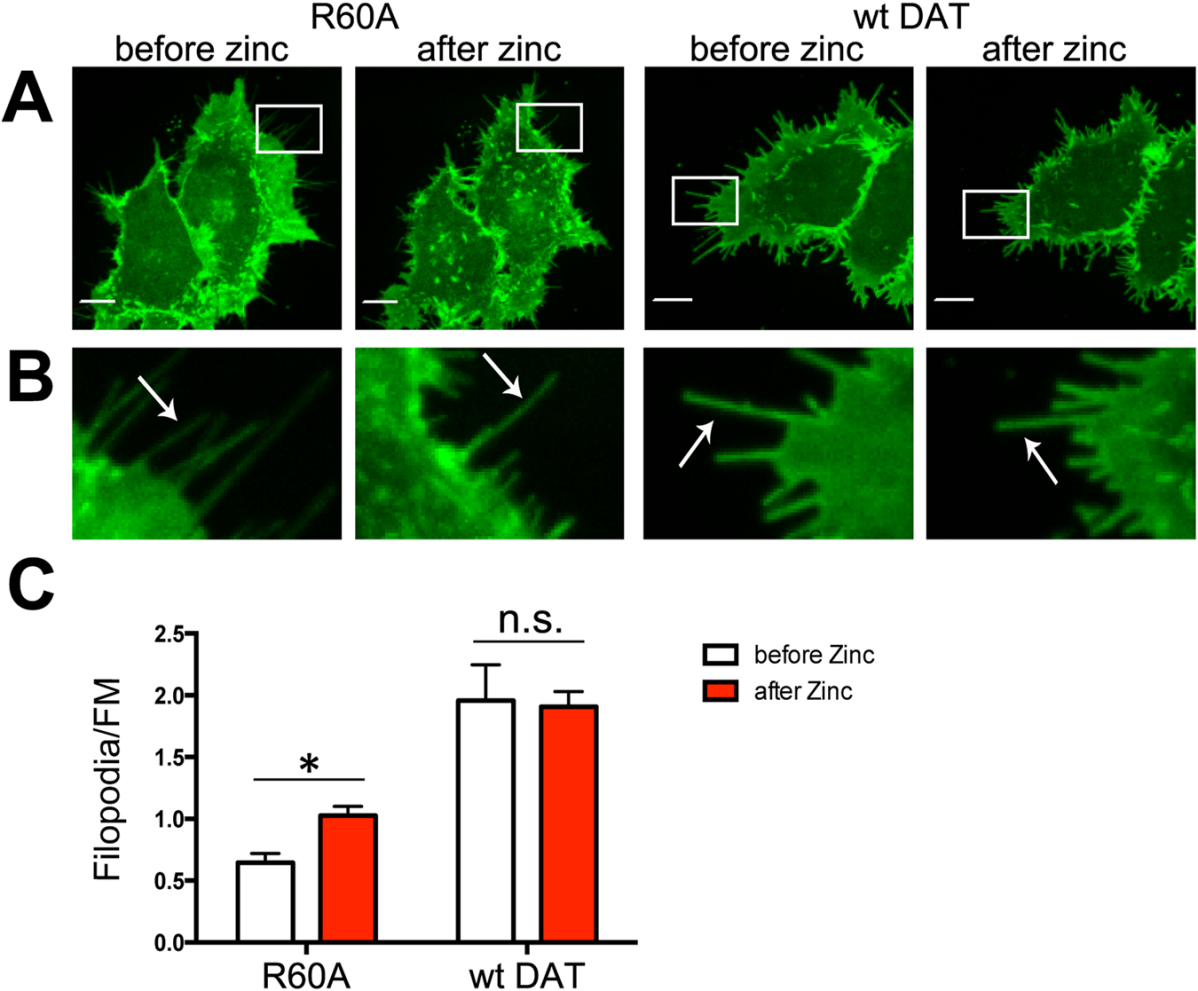
Binding of zinc increases the concentration of R60A in filopodia of HEK293 cells. (**A)** Cells transiently expressing YFP-DAT or the R60A mutant of YFP-DAT were incubated with 10 μM zinc chloride for 30 min at RT. Live-cell imaging was performed through the 515 nm (YFP, *green*) filter channel. Maximal z-projections of 5 consecutive x-y-confocal planes are shown. Scale bars, 10 μm. (**B**) Insets represent high magnification images of the regions marked by the white rectangle in (**A**). Arrows point to examples of peripheral filopodia. (**C**) The filopodia/FM ratios of YFP fluorescence intensities were quantitated in experiments exemplified in (**A**,**B**). Results are shown as mean ± SEM, n = 10. *p < 0.05.

To test whether the stabilization of the OF state by means other than cocaine binding would drive R60A mutant DAT to filopodia, the effect of zinc on DAT localization was tested. Zinc binding to the EC residues in the DAT (e.g. H193, D206, H375 and E396 in the EL2 and EL4 loops) has been reported to shift its conformational equilibrium towards the OF state^33, 34^. Binding of zinc was shown to partially rescue the uptake activity of the R60A mutant, presumably, by stabilizing the OF conformation of this mutant^13^. Therefore, we examined if zinc increased the concentration of the R60A mutant in filopodia. Because H193 was mutated to tyrosine in YFP-HA-DAT to incorporate the HA tag, these experiments were performed in wt and mutant YFP-DAT lacking the HA tag. HEK 293 cells transiently expressing wt YFP-DAT or the YFP-DAT R60A mutant were imaged before and after incubation with 10 μM zinc chloride for 30 min. The filopodia/FM ratio of YFP-R60A fluorescence increased by ~40% after zinc treatment, whereas that of wt YFP-DAT did not change (Fig. 7). These results provided a cocaine-independent evidence that shifting an equilibrium of the DAT conformational state towards the OF conformer increases the concentration of R60A in filopodia.

### AMPH reduces DAT density in filopodia

**Figure 8:**
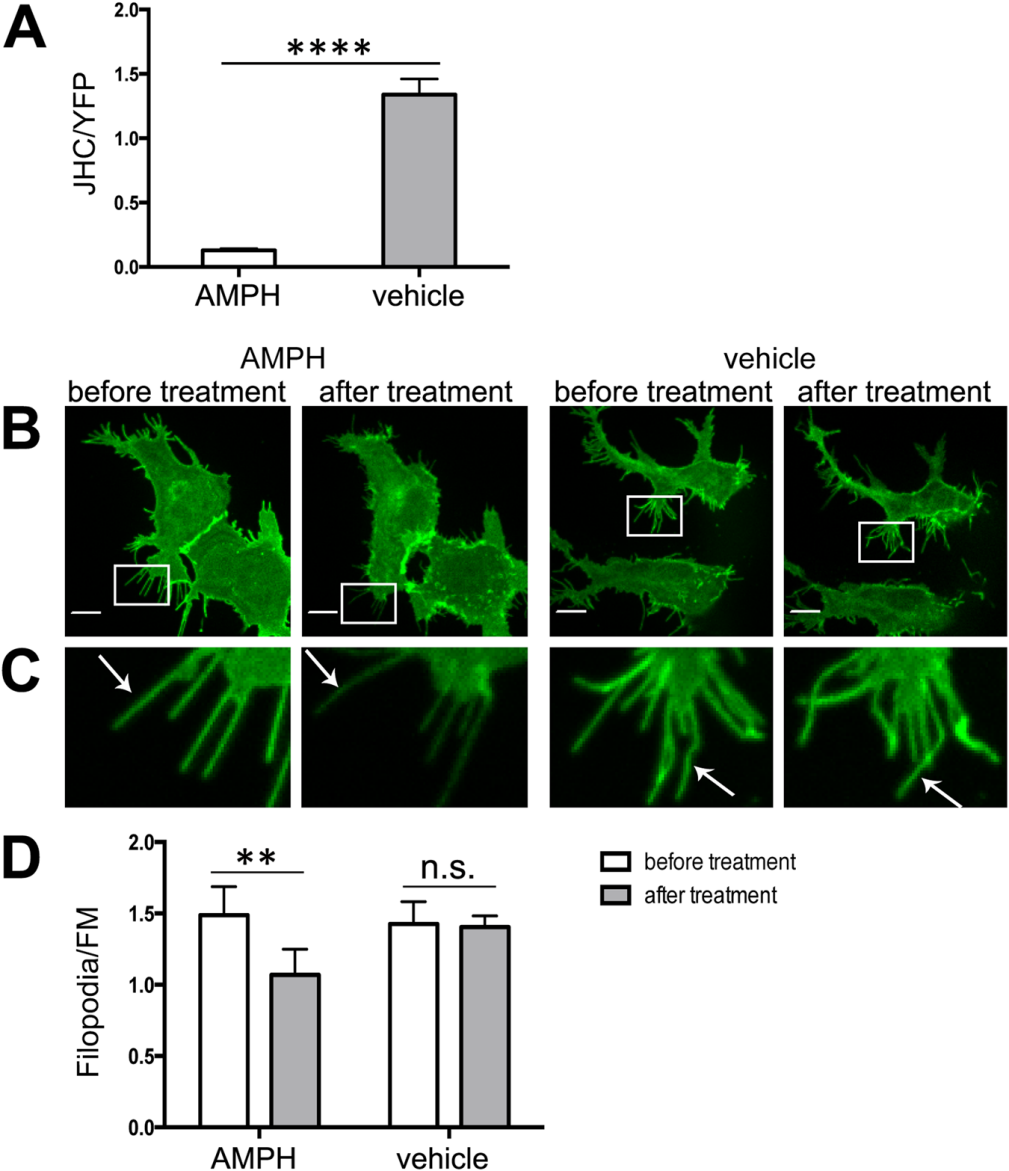
AMPH treatment reduces the concentration of wt DAT in the filopodia of HEK293 cells. (**A**) Cells transiently expressing YFP-HA-DAT were treated with AMPH or vehicle (control) at 37°C for 1 hr. Cells were then incubated with 100 nM JHC1-64 for 10 min. Images were acquired through 515 nm (YFP, green) and 561 nm (JHC1-64, red) filter channels. The ratio of JHC1-64 and YFP fluorescence intensities (JHC/YFP) was calculated for individual cells. Results are shown as mean ± SEM, n = 5. ****p < 0.0001. (**B**) Cells transiently expressing YFP-HA-DAT were incubated with 10 μM AMPH or vehicle in KRH at 37 °C for 30 min. Live-cell imaging was performed through 515 nm (YFP, green) filter channel. Maximal z-projections of 5 consecutive x-y-confocal planes are shown. Scale bars, 10 μm. (**C**) Insets represent high magnification images of the regions marked by the white rectangle in (***B***). Arrows point on examples of peripheral filopodia. (**D**) The filopodia/FM ratios of YFP fluorescence intensities were calculated in experiments exemplified in (**B**,**C**). Results are shown as mean ± SEM, n = 8. **p < 0.01.

In Figs 1, 2, 3, 4 and 7, we demonstrated that stabilizing an OF state of the R60A mutant drives the enrichment of the mutant in the filopodia. To test whether the reverse change, such as an alteration in the conformation of wtDAT favoring an IF state, affects transporter redistribution in the plasma membrane in the opposite direction, HEK293 cells expressing YFP-HA-DAT were treated with AMPH. AMPH is transported by DAT into the cell where it activates signaling pathways leading to phosphorylation of the amino-terminus of DAT, which in turn results in the reverse substrate transport activity of DAT (efflux) and which has been attributed to a shift in the equilibrium distribution of DAT conformation towards its IF state^14, 35, 36^. HEK293 cells transiently expressing YFP-HA-DAT that were treated with AMPH for 1 hour at 37°C did not efficiently bind JHC1-64 as compared with the vehicle-treated cells (Fig. 8A), indicating that the OF conformation of DAT was indeed disrupted after AMPH treatment. Quantification of the Filopodia/FM ratio showed that the density of YFP-HA-DAT in filopodia was decreased by the 30-min AMPH treatment at 37°C (Fig. 8B–D). These results further supported the close relationship between the stabilization of the OF state of DAT and its propensity to target DAT to filopodia.

## Discussion

In this study, we utilized several approaches to control the conformational state of DAT molecules and used quantitative live-cell imaging to demonstrate that the OF state of DAT promotes its targeting to the areas of the plasma membrane with high curvature such as filopodia. In most experiments, we used as a model the R60A mutant of DAT that does not transport substrate and is not concentrated in filopodia^13, 23^, but unlike another non-functional DAT mutant, W63A, is capable of binding cocaine and its analogs. The ability of R60A to bind cocaine and its analogs was demonstrated using JHC1-64 (Fig. 1) and by simulations (Figs 5 and 6). We also demonstrated that this binding drives the R60A, but not the W63A mutant, to filopodia. Present docking simulations (Fig. 6), in accord with previous simulations^11, 14^, suggested that cocaine binding tends to stabilize the OF state of wt DAT or R60A, but not W63A. In agreement with these predictions, and the indications that the OF state favors the targeting of filopodia, increased targeting of R60A to filopodia was observed when an alternative approach of stabilizing the OF state of R60A by zinc binding was used (Fig. 7). The molecular mechanisms of the zinc effect on DAT conformation are not fully understood, although the hypothesis of “correcting” DAT conformation by zinc is supported by the rescue of the substrate uptake activity of the R60A mutant by zinc^13^. The latter observation also demonstrates that DAT localization in filopodia or other outward-curved membrane regions does not interfere with the functionality (e.g. OF/IF state transition kinetics) of DAT.

Binding of cocaine or JHC1-64 did not change the distribution of wt DAT between non-filopodial and filopodia domains of the plasma membrane in our experiments (Figs 2–4). This result is consistent with the original studies where JHC1-64 did not change the surface expression and endocytosis of DAT^31^. It is possible that the bulk of the transporter is present in the OF state in the absence of substrates, and therefore, stabilizing the OF state by cocaine or JHC1-64 does not significantly increase the pool of the wt DAT OF conformer. Indeed, wt DAT is significantly enriched in filopodia, cell edges, and cell-connecting nanotubes at steady-state^24^, and the data in Figs 1 and 2 show that JHC1-64-bound wt DAT (OF state) is accumulated in these regions of the membrane with high curvature. On the other hand, it has been shown using cell surface biotinylation that cocaine increases the plasma membrane concentration of wt DAT ^37^. Such up-regulation of surface DAT at the cell surface could be due to increased accumulation of cocaine-occupied DAT in filopodia, where DAT is proposed to be retained from endocytosis^23^. It is possible that the magnitude of the effects of cocaine on the subcellular distribution of DAT is dependent on total cellular levels of DAT, the relative size of intracellular pool of DAT, and the sensitivity of the DAT detection method.

Additional evidence in support of the hypothesis of the filopodial localization of the OF conformer of DAT was obtained using cell treatment with AMPH that decreased the density of wt DAT in filopodia (Fig. 8). The molecular details of the AMPH effect on the DAT conformational state are not defined, although AMPH, like DA, drives a structural transition toward the IF state of DAT^7, 10, 11, 14^. AMPH activates CaMKII leading to phosphorylation of the amino-terminus of DAT, which is proposed to alter DAT conformation in favor of the substrate binding to the IC site and the reverse transport (efflux)^36^. Re-distribution of a pool of DAT from filopodia to the non-filopodial membrane by AMPH detected by quantitative image analysis is consistent with the ability of AMPH to induce DAT endocytosis^38^, given the surface-retention function of the filopodia localization of DAT^23^.

Based on our previous observations and findings in the present study, we propose a hypothetical model describing the regulation of DAT localization in the membrane by its molecular conformational state (Fig. S3). An atomic structure of DAT in the OF state shows that the TM core of the molecule has a concave shape with the diameter of the cytoplasmic interface of the core smaller than that of the EC interface^6^. Therefore, in the OF state wt DAT or cocaine-bound R60A mutant are stabilized in outward-invaginated (convex-shaped) membranes such as filopodia (Fig. S3). When DAT concentration is high, local membrane crowding by DAT molecules may induce outward bending of the membrane, thus initiating filopodia formation as shown in our previous study^24^. DAT mutants, in which mutations disrupt intramolecular interactions required to stabilize the OF conformer, or amino-terminally phosphorylated wt DAT, do not maintain a concave shape, and therefore have a lower propensity to occupy curved membranes and do not concentrate in filopodia (Fig. S3). Oligomerization of DAT in intact cells has been demonstrated^39, 40^, and future studies will address whether oligomerization, that would increase the “concaveness” in the DAT multimer, is an additional factor in targeting DAT to the membrane regions with high curvature (Fig. S3A). Finally, another potential mechanism of stabilizing DAT in the filopodia may involve DAT interactions with actin-associated proteins or lipids enriched in filopodia. DAT interaction with phosphatidylinositol-4,5-bisphosphate (PIP2), a lipid that is enriched in some types of filopodia^41^, has been proposed^42^. However, deletion of the putative PIP2 binding site in the amino-terminus of DAT (amino acid residues 1–36) did not abolish the DAT enrichment in filopodia^23^.

The ability of proteins with domains that have intrinsic convex or concave shapes to sense high-curvature membrane regions, concentrate in these regions, or contribute to membrane bending is well established^43^. Membrane-associated cytosolic proteins with I-BAR domain such as IRSp53 and MIM (missing in metastasis) act as scaffolds to induce membrane bending during filopodia formation^27, 29, 44^. The relationship between transmembrane proteins and membrane curvature or re-modeling is much less understood, and only few examples have been described. Potassium channel KvAP has been demonstrated to be enriched in membrane nanotubes formed from giant unilamellar vesicles *in vitro* ^45^. The chemoreceptor TlpA oligomers of *Bacillus subtilis* are localized to highly curved membranes and this localization is affected when the convex shape of the receptor is disrupted by mutations^46^. Assembly of ATP synthase dimer rows enables the formation of highly curved ridges in mitochondrial cristae in yeast^47^. To our knowledge, the present study of DAT highlights the first example of the regulation of membrane curvature sensing and submembrane distribution of a mammalian multi-membrane-spanning protein by its conformational states and their transitions. Such regulation may play an important role in DAT function in the brain. Our previous studies demonstrated the localization of endogenous DAT in filopodia of post-natal dopaminergic neurons^22, 24^. DAT is also readily detected in the filopodia of dopaminergic axons in acute slices of the adult mouse striatum (data not shown). A large fraction of filopodial DAT in neurons or heterologous cells is shown to be immobile, and the disruption of the filopodial localization of DAT facilitates its endocytosis^22, 23^. Therefore, we propose that the accumulation of immobile DAT in filopodia of growing axons in developing neurons and in the fine axonal network of the adult striatum may serve to maintain a pool of functional plasma membrane DAT, which is protected from excessive endocytosis and down-regulation that may be caused by external factors or aberrant intracellular signaling associated with various pathologies of the dopamine system.

## Methods

### Reagents

Cocaine hydrochloride, D-amphetamine hemisulfate salt and ZnCl_2_ were from Sigma-Aldrich (St. Louis, MO). JHC1-64 was synthesized in the Medicinal Chemistry Section, NIDA-IRP as previously described^30^. Restriction enzymes are from New England Biolabs (Ipswich, MA). All other chemicals were from Thermo Fisher Scientific (Pittsburgh, PA) or Sigma-Aldrich.

### Plasmids

Yellow fluorescent protein (YFP) and hemaglutinin epitope (HA) tagged human DAT (YFP-HA-DAT, Addgene plasmid # 90244) and mutant versions of YFP-HA-DAT (R60A and W63A, Addgene plasmids # 90245 and #90246, respectively) were described previously^23, 48^. RFP-HA-DAT (Addgene plasmid # 90265) was also previously described^24^. To generate R60A mutation in the template of YFP-DAT (no HA tag, Addgene plasmid # 90228)^39^, the 1622 bp DAT sequence (begins with bp 242 in DAT cDNA) in YFP-HA-R60A was replaced by the corresponding sequence from the YFP-DAT construct using PflMI (5’ end) and SmaI (3’ end) restriction sites. The R60A mutation in YFP-DAT (Addgene plasmid # 90247) was confirmed by sequencing.

### Cell culture and transfections

Human HEK293 cells (Invitrogen # R70507) were grown in DMEM with 10% fetal bovine serum (FBS). Porcine aortic endothelial (PAE) cells were originally obtained from Dr. B. Westermark (University of Uppsala, Sweden) and used in our previous studies^23, 24, 39, 48^. PAE cells were grown in F12 medium with 10% FBS. The cells were transiently transfected with plasmids using Effectene kit (Qiagen, Valencia, CA) according to the manufacturer’s protocol. After transfection, HEK293 cells were grown on Poly-D-Lysine (Sigma-Aldrich) coated 18 mm glass coverslips and PAE cells were grown on coverslips without coating. Cells were used for imaging 2 days after transfection.

### Fluorescence microscopy

Spinning disk confocal imaging system equipped with EM-CCD camera and environmental chamber controlled by SlideBook software (Intelligent Imaging Innovation, Denver, CO) was described previously^24^. 63x oil immersion lens was used (207 nm/pixel). For JHC 1-64, cocaine and zinc binding experiments, cells were imaged in Dulbecco’s Phosphate Buffered Saline (DPBS) (plus 1 mM CaCl_2_, 0.5 mM MgCl_2_ and 5 mM D-glucose) at room temperature (RT). For AMPH treatment experiment, cells were imaged in Krebs Ringer HEPES  solution (KRH, with 140 mM NaCl, 5 mM KCl, 2 mM CaCl_2_, 1 mM MgCl_2_, 5.5 mM HEPES and 1 mM D-glucose, pH 7.4), at 37°C, humidity and 5% CO_2_. A z-stack of 15-20 confocal images at 400 nm z-steps was acquired through 515 nm (YFP) or 561 nm (RFP and JHC1-64) laser channels using a single dichroic. All images were captured using the same exposure time (100 ms) and all other image acquisition parameters. Gamma was set on “1” in all images used for presentation in all figures of the manuscript.

### Image analysis

Images were analyzed using SlideBook6 software. The use of ﻿an ﻿EM-CCD camera allows high linearity of fluorescence detection within the full intensity range. Background was subtracted in each image. To quantitate JHC1-64 binding, segmental mask was generated to select YFP-containing voxels in each individual cell. Mean fluorescence intensities (in arbitrary linear units of fluorescence intensity) of JHC1-64 and YFP were calculated in the mask, and the ratio of JHC1-64 and YFP fluorescence, designated as JHC/YFP, was used to estimate the apparent density of the JHC1-64:YFP-HA-DAT complexes. YFP intensities were corrected for photobleaching whereas no significant photobleaching of JHC1-64 fluorescence was observed during time-course imaging.

To quantitate the ratio of JHC1-64 to YFP-HA-DAT fluorescence intensities in filopodia versus non-filopodial regions of the plasma membrane (FM, flat membrane), 5 xy-confocal planes from the z-stack that include most of the peripheral filopodia and cell-bottom plasma membrane were used for quantification. Peripheral filopodia were selected manually using SlideBook mask pencil tool in each z-plane (Mask 1). A mask containing all YFP-containing voxels in the cell was also generated using automated segmentation (Mask 2). The overlapping voxels of Masks 1 and 2 were then selected to generate Mask 3 (filopodia) to minimize the error of the manual selection of YFP-positive voxels. Ten or more representative small regions of FM in the periphery of the cells were manually selected to generate FM Mask 4. This mask typically included fluorescence of both bottom and top cell surface due to a limited z-axis resolution. Mean fluorescence intensities (in arbitrary linear units of fluorescence intensity) of JHC1-64 and YFP in Mask 3 and Mask 4 were then calculated in each cell, and the JHC/YFP ratio was used to estimate the relative density of JHC1-64:YFP-HA-DAT complexes in filopodia (Mask 3) and non-filopodial plasma membrane (Mask 4).

To quantitate the relative density of YFP-HA-DAT fluorescence in filopodia and non-filopodial FM regions of the plasma membrane, filopodia (Mask 3) and FM (Mask 4) masks were generated from 5 xy-confocal planes from full z-stack of images as described above for experiments with JHC1-64. The mean YFP fluorescence intensities of filopodia Mask 3 and FM Mask 4, and their ratio (Filopodia/FM) were calculated in individual cells.

In cells co-expressing YFP- and RFP-tagged DATs, filopodia (Mask 3) and FM (Mask 4) masks were generated as described above, and the ratio of mean YFP and RFP fluorescence intensities in Mask 3 to Mask 4 were calculated for each individual cell to obtain filopodia/FM ratio values for YFP-HA-DATs and RFP-HA-DAT, respectively. Furthermore, the values of YFP filopodia/FM ratio were divided by values of the RFP filopodia/FM ratio in each individual cell to obtain an YFP/RFP value that corresponds to the enrichment of YFP-HA-DAT relative to RFP-HA-DAT in filopodia.

### MD simulations of DAT mutants

Atomic structure of human (h)DAT (Q58 to E598) in the outward-facing open (OFo) state was taken from previous studies^11, 14^, and constructed after the Drosophila DAT crystal structure^6^. Structures of W63A and R60A mutants were solved in-silico using VMD^49^, and the mutant simulation systems were prepared following previous approach^11, 14^. Briefly, the TM of each mutant was inserted into the center of the pre-equilibrated POPC (1-palmitoyl-2-oleoylphosphatidyl choline) lipid bilayer. Fully equilibrated TIP3 waters were added to form a box of 104.6 × 104.6 × 150 Å3. Sodium and chloride ions corresponding to a 0.15M solution were added to neutralize the system. The simulation box contained one hDAT mutant, 196 POPC molecules, 0.15 M NaCl solution, summing up to a total of over 140,000 atoms. CHARMM36 force field with CMAP corrections was used for protein, water and lipid molecules^50, 51^. All simulations were carried out using NAMD^52^, following the conventional MD protocol used for the wild type (WT) hDAT^11, 14^. For each mutant, two independent MD runs of 100 ns were performed.

### Docking simulations

Docking simulations were performed with AutoDock^53^ using the OFo conformers, sampled during MD simulations. For each conformer, both cocaine and JHC 1-64^30^ binding poses were analyzed. The 3D molecular structure of JHC 1-64 was generated using Open BaBel^54^. 100 independent docking runs were performed using a Lamarckian genetic algorithm with default parameters, with the maximal number of energy evaluations set to 2.5 × 107. The simulation box was divided into 112 × 112 × 126 grids with a spacing of 0.6 Å. The binding energy was estimated from the weighted average from multiple binding poses of the small molecule at a given site. The ranks of the binding sites were evaluated from the binding affinity calculated using AutoDock^53^ and the docking occupancy (preferably that with the highest occupancy probability over the possible alternate poses).

### Trajectory analysis

VMD^49^ with in-house scripts was used for visualization and analysis. In all MD runs, the C^α^ root-mean-square deviations (RMSDs) of the structure (amino acids R58-E598 excluding the structurally unresolved part of EL2 loop) from initial conformers converged to 2.1 ± 0.4 Å after 30 ns simulations. The EC and IC openings of DATs were assessed based on separation between the EC-exposed (TM1b and TM10 segments), and IC-exposed helical segments (TM1a-TM6b), respectively, using the method described earlier^11, 14^. The majority the WT and R60A conformers adopted an OF open state; but W63A conformers exhibited inward-facing like state, with increase in the IC vestibule and decrease in the EC vestibule.

### Statistical analysis

All data were analyzed using GraphPad Prism 6.0. Two-way ANOVA followed by Bonferroni test was used in Figs 2-4, 7, 8D, S1, S2; and ﻿Student t-test was used in Figs 1, 8A, to compare the difference among experimental groups. Data normality of all data sets was confirmed using the D’Agostino-Pearson test.

### Data availability

The datasets generated in live-cell imaging experiments and MD/docking simulations are available from the corresponding author on request.

## Electronic supplementary material

Supplementary Information
