# Supplementary material for: Targeting of dopamine transporter to filopodia requires an outward-facing conformation of the transporter
Source: Sci Rep. 2017 Jul 14;7:5399. doi: 10.1038/s41598-017-05637-x (PMC5511133; doi:10.1038/s41598-017-05637-x)
Supplement: Supplementary file 1 — Supplementary Information [file 41598_2017_5637_MOESM1_ESM.pdf]

SUPPLEMENTAL INFORMATION

Targeting of dopamine transporter to filopodia requires an outward-facing conformation of the transporter

Shiqi Ma, Mary H. Cheng, Daryl A. Guthrie, Amy H. Newman, Ivet Bahar, and Alexander Sorkin

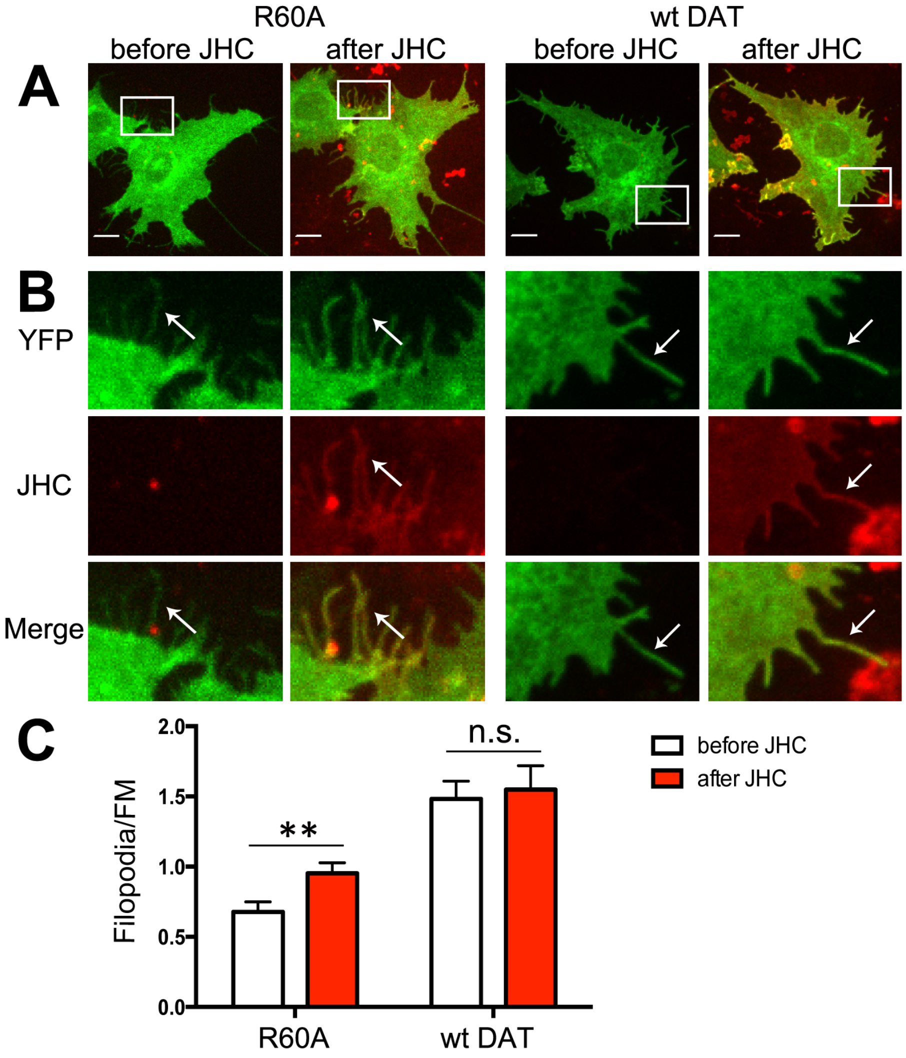

Supplementary Figure S1. Binding of JHC1-64 increases the concentration of R60A in the filopodia of PAE cells.

(A) Cells transiently expressing YFP-HA-DAT or the R60A mutant were incubated with 100 nM JHC1-64 for 30 min at RT. Live-cell imaging was performed through 515 nm (YFP, *green*) and 561 nm (JHC1-64, *red*) filter channels. Maximal z-projections of 5 consecutive x-y-planes are shown. Scale bars, 10μm.

(B) Insets represent high magnification images of the regions marked by the white rectangle in (*A*). Arrows point to representative filopodia.

(C) The filopodia/FM ratios of mean intensities of the YFP fluorescence were calculated as described in “Methods”. Bar graphs represent mean values (± SEM, n=10). **p<0.01.

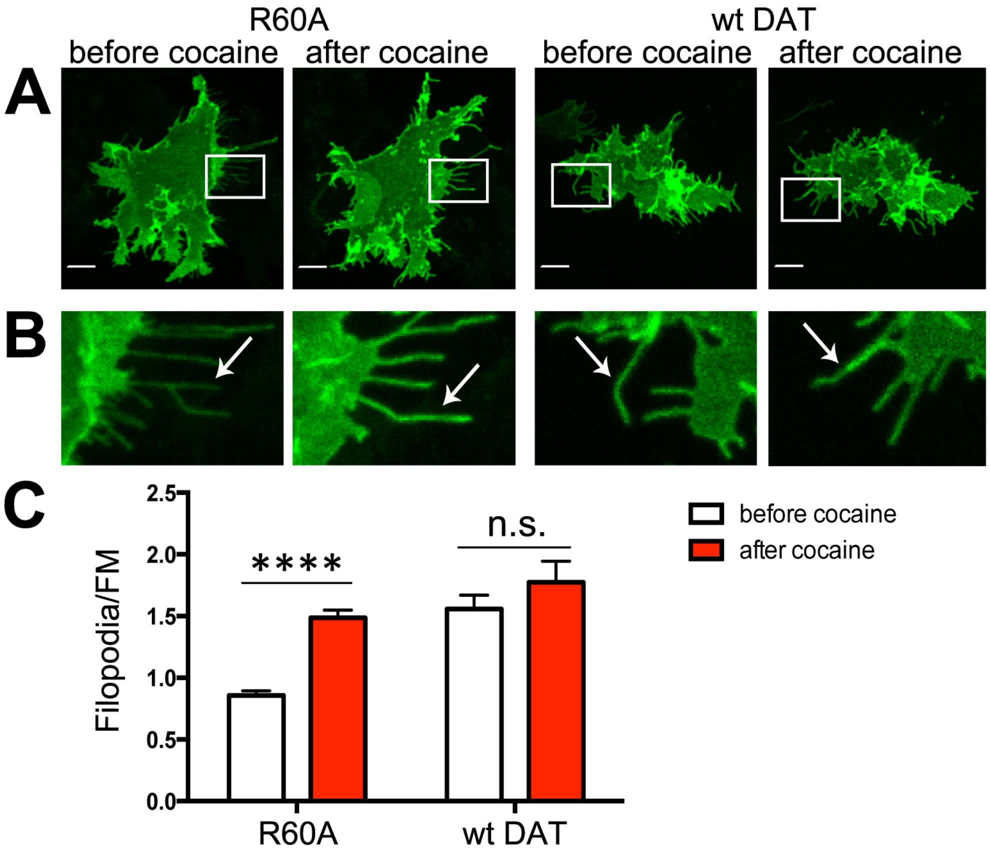

Supplementary Figure S2. Binding of cocaine increases the concentration of R60A in the filopodia of PAE cells.

(A) Cells transiently expressing wt YFP-HA-DAT or the R60A mutant were incubated with 10µM cocaine for 30 min at RT. Live-cell imaging was performed through the 515 nm (YFP, *green*) filter channel. Maximal z-projections of 5 consecutive x-y-confocal planes are shown. Scale bars, 10µm.

(B) Insets represent high magnification images of the regions marked by the white rectangle in (***A***). Arrows point to representative filopodia.

(C) The filopodia/FM ratios of mean intensities of the YFP fluorescence were calculated as described in the "Methods". Results are shown as mean values (± SEM, n=10). ****$p<0.0001$.

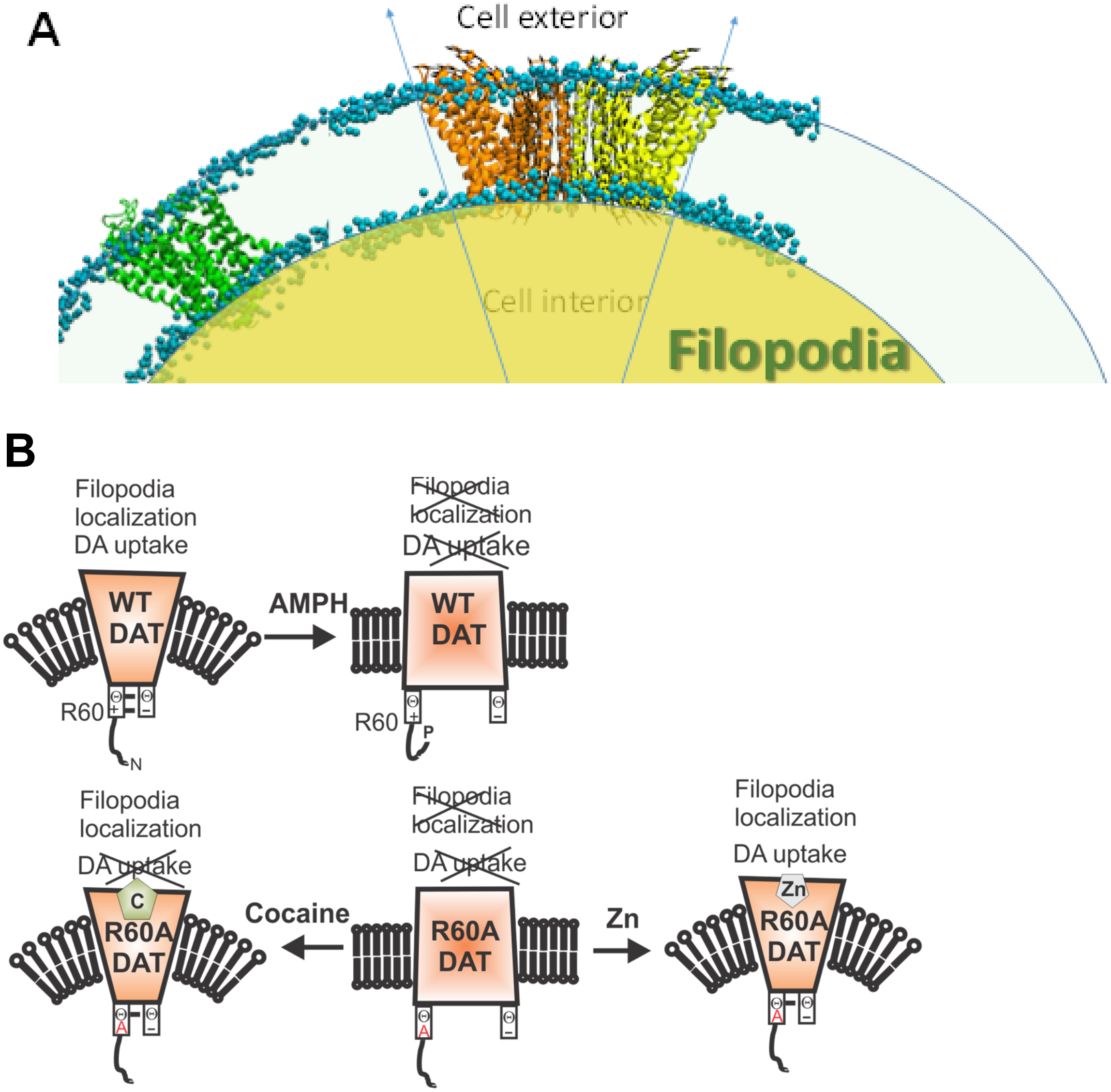

Supplementary Figure S3: Hypothetical model of the regulation of DAT concentration in curved membranes by its conformation.

(A) Illustration of a monomer (green ribbons) and a dimer (orange and yellow ribbons) embedded into filopodia.

(B) The OF conformer of wt DAT tends to accumulate in membrane regions with high curvature like filopodia and cell edges due to the concave shape of the OF conformer. AMPH shifts equilibrium of DAT conformation towards an IF state and thus reduces DAT concentrations in filopodia. Non-functional DAT mutant R60A with disrupted OF conformation is not targeted to curved membranes. Binding of cocaine or zinc to R60A stabilizes its OF state and a convex shape, thus driving R60A to filopodia.
